# Neuroinflammation in the pathogenesis of Alzheimer’s disease. A rational framework for the search of novel therapeutic approaches

**DOI:** 10.3389/fncel.2014.00112

**Published:** 2014-04-22

**Authors:** Inelia Morales, Leonardo Guzmán-Martínez, Cristóbal Cerda-Troncoso, Gonzalo A. Farías, Ricardo B. Maccioni

**Affiliations:** ^1^Laboratory of Cellular and Molecular Neurosciences, Faculty of Sciences, University of ChileSantiago, Chile; ^2^International Center for Biomedicine (ICC)Santiago, Chile; ^3^Department of Neurology and Neurosurgery North, Faculty of Medicine, University of ChileSantiago, Chile; ^4^Department of Neurological Sciences East, Faculty of Medicine, University of ChileSantiago, Chile

**Keywords:** neuroinflammation, Alzheimer disease, microglia, astrocytes, nutraceuticals

## Abstract

Alzheimer disease (AD) is the most common cause of dementia in people over 60 years old. The molecular and cellular alterations that trigger this disease are still diffuse, one of the reasons for the delay in finding an effective treatment. In the search for new targets to search for novel therapeutic avenues, clinical studies in patients who used anti-inflammatory drugs indicating a lower incidence of AD have been of value to support the neuroinflammatory hypothesis of the neurodegenerative processes and the role of innate immunity in this disease. Neuroinflammation appears to occur as a consequence of a series of damage signals, including trauma, infection, oxidative agents, redox iron, oligomers of τ and β-amyloid, etc. In this context, our theory of Neuroimmunomodulation focus on the link between neuronal damage and brain inflammatory process, mediated by the progressive activation of astrocytes and microglial cells with the consequent overproduction of proinflammatory agents. Here, we discuss about the role of microglial and astrocytic cells, the principal agents in neuroinflammation process, in the development of neurodegenerative diseases such as AD. In this context, we also evaluated the potential relevance of natural anti-inflammatory components, which include curcumin and the novel *Andean Compound*, as agents for AD prevention and as a coadjuvant for AD treatments.

## Introduction

Alzheimer’s disease (AD) is the most common type of dementia and usually affects people over 65. AD is characterized by accumulation of intra and extracellular protein aggregates. Extracellular deposits correspond to amyloid plaques, mainly composed of a 39 to 42 amino acids peptide called β-amyloid (Aβ), generated by proteolytic cleavage of amyloid precursor protein (APP) by beta and gamma secretases. Intracellular protein deposits are named to as neurofibrillary tangles (NFTs) and are composed of hyperphosphorylated τ protein assembled in oligomeric structures called paired helical filaments (PHF). PHF and NFT deposition causes loss of synaptic function and finally neuronal death (Giannakopoulos et al., 2003). This process of neurodegeneration self amplifies when τ aggregates are released into the extracellular environment, since there is an important body of evidence supporting neurotoxicity of τ aggregates (Neumann et al., 2011). Among that evidence we can mention cell culture studies showing that overexpression of τ alters cell morphology, delays cell growth, and changes the distribution of organelles that are transported through the axis by motor axonal microtubule-associated proteins (reviewed in Cambiazo et al., 1995). Moreover, in transgenic mice overexpressing a human τ isoform with four repeated sequences, axonal degeneration develops in the brain and dorsal root ganglia, and is related to accumulation of neurofilaments (Spittaels et al., 1999).

Numerous therapeutic targets to eradicate the AD or lessen its symptoms have been used through years of research. Among the most studied mechanisms for AD treatment is beta amyloid clearance by passive or active immunization but this methodology has been unsuccessful and even deleterious so far (Citron, 2010). On the other hand, neuroinflammation in central nervous system (CNS) appears as a central event in AD pathophysiology. There are promising targets for AD treatment in relation to neuroinflammation and the mechanisms of cross talks between microglia and neurons (Fernández et al., 2008; Morales et al., 2010; Maccioni, 2011; Neumann et al., 2011). In this review, we will address on how neuroinflammatory processes are directly related to cognitive decline and neurodegenerative processes. Moreover, we will describe the implications of the involvement of both astrocytes and microglia in both the inflammatory and neuroimmunomodulatory processes.

## Neuroinflammation

The inflammatory response is almost always a secondary response caused by an initial event after another, like the response to trauma or infections. However, this means it is a central mechanism in the neurodegenerative processes. It is this secondary response that will ensue and probably cause a greater loss of neurons over time as compared to the initial injury (Akiyama et al., 2000). Inflammation plays a key role as a driving force that can modulate the development of various neuropathologies.

Currently the term “neuroinflammation” is used to describe the inflammatory response originated in the CNS after suffering an injury, where there is an accumulation of glial cells. Particularly astrocytes and microglia responses converge immediately after the injury occurs. In this process, cellular and molecular immune components such as cytokines, complement and pattern-recognition are contributing players, and they can lead to the activation of the glial cells, i.e., microglia and astrocytes.

Innate immunity is the first line of defense of the organism against different pathogens. Among the components of the response we can mention pattern-recognition receptors (PRRs), such as toll-like receptors (TLRs), nucleotide-binding, Scavenger receptors (SRs), among others. These receptors recognize not only exogenous pathogen-associated molecular pattern (PAMP) but also endogenous modified molecules called damage-associated molecular pattern (DAMP). Throughout the body, the innate immune system launches inflammatory and regulatory responses via PRRs, phagocytes (macrophages), complement system, cytokines, and chemokines in order to counteract infection, injury, and maintain tissue homeostasis. Agents involved in innate immunity, are directly related to agents involved in the development of neuroinflammation. Cells of the CNS such as neurons, astrocytes, and microglia along with pattern recognition receptors, cytokines, chemokines, complement, peripheral immune cells, and signal pathways constitute the basis for neuroinflammation (Shastri et al., 2013).

**Figure 1:**
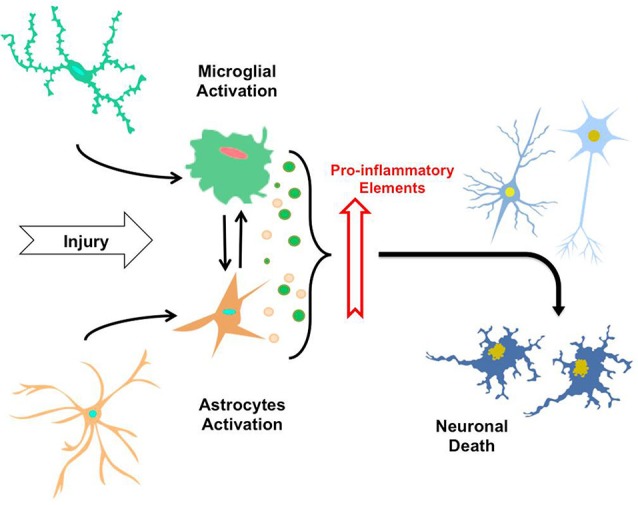
**The neuroinflammatory process**. By sensing signals of damage or injury, astrocytes and microglia suffer a gradual activation process, leading to morphological changes and secretion of pro-inflammatory elements (i.e., cytokines, cytotoxic elements, ROS). Thus, the constant exposure of astrocytes and microglia to factor causing injuries and secretion of these elements induce mutual activation of microglial cells and astrocytes, along with neuroinflammatory process that eventually trigger neuronal death.

An acute inflammatory response in the CNS is caused by the immediate and early activation of the glial cell in response to noxious stimuli, which is basically a defensive response that leads to repair of the damaged area. But, if the “stimulus” remains persistent in time, an inflammatory condition develops, causing a phenomenon of cumulative damage over time due to the chronic inflammatory reaction (Streit et al., 2004). All these events precede and cause neuronal degeneration, generating complex interactions and feedback loops between glial and neuronal cells, leading to cell damage and to the development of a neurodegenerative disease. Thus, neuroinflammation has beneficial or deleterious results in the brain mainly depending on the duration of the inflammatory response (Figure 1).

It has been possible to associate a number of neurodegenerative disorders of the CNS to neuroinflammatory events, for example, based on the appearance of high levels of several pro-inflammatory cytokines: AD, Parkinson’s disease (Ferrari et al., 2006), Huntington’s disease (Hsiao et al., 2013), multiple sclerosis (MS), amyotrophic lateral sclerosis (ALS; Kassa et al., 2009) among others. In all these diseases neuropathological and neuroradiological studies have been performed providing evidence that neuroinflammatory responses could start prior to a loss of neuronal cells. In this regard, increasing evidence has been obtained on the role of certain cytokines in the direct activation of the cellular cascade leading to neurodegeneration and AD (Table 1). It would be interesting to identify as correlate the neuroinflammation levels that leading to release of these cytokines, which have neurotoxic effects and are involved in the progression of this disorder pathophysiological process (Frank-Cannon et al., 2009).

**Table 1:** **Pro-inflammatory elements secreted by astrocytes and microglia during the process of neuroinflammation**.

**Pro-Inflammatory Elements**	**Effect**
Chemokines	Dysfunction, apoptosis and necrosis of neuron, microglia and astrocytes
IL-1β, IL-6, IL-12 INF-γ, TNFα	Astrocytes and microglia activation; dysfunction, apoptosis and necrosis of neuron, microglia and astrocytes
NO, ROS, O_2−_	Oxidative stress in cells; dysfunction, apoptosis and necrosis of neuron, microglia and astrocytes

Specifically in AD, it has been demonstrated that there is a high expression of inflammatory mediators in the vicinity of Aβ peptide deposits and neurofibrillary tangles, which in turn are associated with areas of high neurodegeneration (Akiyama et al., 2000); exemplifying the relationship between neuroinflammation and neurodegeneration (Figure 1).

Moreover, epidemiological studies have established a link between chronic use of non-steroidal anti-inflammatories (NSAIDs) and reduced risk of AD (Vlad et al., 2008). These studies reported that the use of long-term NSAID has a protective effect against the development of the disease, delaying the onset of the symptoms or reducing the risk of its occurrence. Although the mechanism behind this phenomenon is still unknown, some hypotheses are inclined to the effects of these anti-inflammatory on the regulation of COX-1 and COX-2 protein, whose levels are elevated in individuals with AD (Vlad et al., 2008). It has also been observed that the regulation and blockade of the COX-1 in microglia, by effect of NSAIDs induced an improvement in the symptomatology of AD (McGeer and McGeer, 2007). Results in transgenic animal models of AD, show that NSAIDs reduce in a dose-dependent manner behavioral deficits and the population of activated microglia (McGeer and McGeer, 2007).

Comparative analyses performed in the brains of cognitively normal patients chronically using NSAIDs over age versus those not using NSAIDs revealed no changes in the appearance of senile plaques, but there was a 3-fold decrease in the number of activated microglia in the brains of chronic users of NSAIDs (McGeer and McGeer, 2007). AD patients who used NSAIDs compared with another group of patients who did not use NSAIDs, showed a significantly slower progression of disease (Rich et al., 1995). These findings suggest that the protection provided by the chronic use of NSAIDs in patients with AD may partly be derived from the attenuation of microglial activation.

Lim et al. have conducted studies of cerebral amyloidosis in transgenic mouse models, and gave evidence of ibuprofen effect on amyloid plaque deposition (Lim et al., 2000). After 6 months of treatment with ibuprofen, amyloid plaque deposits were reduced significantly in 10 months old Tg2576 transgenic mice. Also there was a reduction in markers of astroglial and microglial activation (Lim et al., 2000). Moreover, in double transgenic animals ibuprofen reduced microglial activation and decreased the number of amyloid deposits (Yan et al., 2003; Heneka et al., 2005). In addition, Choi et al. demonstrated that the treatment of 20-month-old triple transgenic AD (3 X Tg-AD) mice with the COX-1 inhibitor SC-560, significantly improved memory deficits and reduced amyloid deposition and τ phosphorylation in the hippocampus (Choi et al., 2013). An explanation is that the mice SC-560 treatment alters the phenotype of activated microglia, reducing the expression of pro-inflammatory factors. Authors postulated that this changes in microglial cells may play a role in the reduction of amyloid charge and τ pathology and in rescuing impaired memory in aged 3 X Tg-AD. As stated above, there is important evidence that early treatment in transgenic animals with NSAIDs may reduce neuroinflammation and Aβ peptide deposition in the brain (Jantzen et al., 2002). In spite of that it cannot be overlooked that not all results have been favorable, for example, in transgenic mice models of AD, COX-2 selective inhibitors failed to reduce the inflammatory reaction and showed an increase in the appearance of Aβ42 peptide (Kukar et al., 2005). Recent findings suggest that alterations in microglia and the production of cytokines and chemokines may be an early feature that precedes Aβ deposition in a mouse model of AD (Varvel et al., 2009). This early microglial activation may well play a role in the appearance of vulnerable neuronal populations, similarly to the situation in AD.

On the other hand, clinical trials on the effects of NSAIDs treatments of cognitive decline in Alzheimer’s disease patients did not provide clear-cut results, data varied depending on the cognitive test used. For example, results of trials with Naproxen indicate that this NSAID attenuate cognitive decline, but accelerated cognitive decline in fast decliner patients. Conversely, Celecoxib (another NSAID) appears to have similar effects, but attenuated changes in fast decliners (Leoutsakos et al., 2012). Thus, it is premature to make clinical recommendations, but the findings to date, open several potential avenues of research, and possibly the clinical trials should be replicated in one or more large observational studies.

In this context, we can conclude that some NSAIDs are able to reduce the inflammatory response caused by microglial and astroglial cells, but some others are not as effective or may even produce opposite results. This suggests that microglia have different responses after exposure to different types of NSAIDs according to specific mechanism of action of the molecule and also to the source of the primary insult that induces the onset of an inflammatory response. It is also plausible that the response may vary during the course of a given therapy (Jantzen et al., 2002).

## Astrocytes

Astrocytes are the most abundant glial cells of the nervous system and constitute about 25% of the cerebral volume (Tower and Young, 1973). They have multitude of functions: (i) inducing the formation of neuronal synapses and influencing their development (synaptogenesis); (ii) formation and maintenance of blood-brain barrier; (iii) neurotransmission; (iv) metabolic regulation; (v) ion balance maintenance, and finally (vi) as a component of the “tripartite synapse” model of neurotransmission, in this model of neurotransmission, synapse consists of three functional elements: pre-and postsynaptic neurons and surrounding astrocytes. Then in addition to communication between neurons, there is a bidirectional communication between neurons and astrocytes, implying a predominant role of glial cells in the physiology of the nervous system (Matyash and Kettenmann, 2009; Chaboub and Deneen, 2013). Astrocytes play a key role in the development of the nervous system, since the growing of axons is guided to the target by molecules derived from astrocytes, such as tenascin C and proteoglicans (Powell and Geller, 1999).

Astrocytes are actively involved in synaptogenesis, not only during development but also after CNS injury. In 1997, studies conducted by Pfrieger observed that retinal ganglion cells synaptic activity was 100 times major in the presence of astrocytes (Pfrieger and Barres, 1997). This increase in synaptic activity mediated by astrocytes is precisely due to the increased number of synapses, which is seven times higher in retinal ganglion cells cultured with astrocytes in the absence of astrocytes (Ullian et al., 2001). This increase in the number of synapses is mediated by a matrix-associated protein named thrombospondin (Christopherson et al., 2005; Risher and Eroglu, 2012), which belong to a family of five homologous proteins, and at least four of them are expressed in these cell types during development and after brain damage, inducing synaptogenesis. These proteins induce ultrastructurally normal synapse formation, both presynaptic and postsynaptic (Barres, 2008).

On the other hand, the metabolic support given by astrocytes, provides active neurons with metabolic substrates through a glucose-lactate shuttle. Increased neuronal activity leads to an increase in glutamate release, which in turn activates astroglial Na^+^-dependent glutamate transporters, thus increasing cytosolic Na^+^ concentration in astrocytes. In turn, increased Na^+^ stimulates glycolysis and lactate synthesis. The lactate is subsequently transported to neurons through specific transporters (Magistretti, 2009). The astrocytes are involved in the maintenance of homeostasis of brain neurotransmitters, being of particular importance for homeostasis and turnover of glutamate by being the main sink of glutamate in the brain. From the bulk of glutamate released during synaptic transmission, several studies have shown that only a minimum percentage of glutamate (∼20%) accumulates in the neurons, while the largest amount of this neurotransmitter is absorbed by perisynaptic astrocytes. This process of eliminating extracellular glutamate by astrocytes, it is extremely critical to prevent excitotoxicity (Verkhratsky and Kirchhoff, 2007).

Numerous records show that astroglial cells possess highly important functions within the brain. However, pathological modifications of astrocytes have been associated with several neurodegenerative disorders. These include ALS, MS, AD, Parkinson’s disease, Alexander’s disease, epilepsy and Rett syndrome (Okabe et al., 2012). Pathological astrocytes observed in the brains of patients with dementia were initially analyzed by Alois Alzheimer, which found abundant glial cells in the neuritic plaques. Subsequent studies have confirmed that this is a morphological characteristic of reactive astrogliosis in AD that can be found both in brain tissue of patients with AD, and transgenic animal models (Verkhratsky et al., 2010). In studies of post mortem brain tissue from patients with AD a generalized astrogliosis—manifested by cell hypertrophy and an increase in the expression of Glial fibrillary acidic protein (GFAP) in astroglial S100B protein—can be found (Verkhratsky et al., 2010). A more detailed analysis of astrogliosis in brains obtained from elderly patients (with and without AD confirmed) has shown a correlation between the degree of astrogliosis and cognitive impairment. However, a direct relation between changes in astrocytes and increase in senile plaques has been found (Simpson et al., 2010). Morphological data show that reactive astrocytes associate with some Aβ plaques, but not with all of them, while astrogliosis can also been found in areas without Aβ deposits. This may result from the fact that astrocytes may also respond to other pathological factors in the ageing brain (Simpson et al., 2010). In the meantime, no significant difference was found in the expression of GFAP in brain tissue samples from patients with and without dementia (Wharton et al., 2009). Furthermore, it has been shown that fragments of Aβ promote marked inflammatory response in the brain, causing the synthesis of different cytokines and proinflammatory mediators (Lim et al., 2013). Within this inflammatory response, astrocytes express a repertoire of receptors for inflammatory cytokines (IL-1β and TNFα), chemokines and damage signals (including TLR ligands) (Krasowska-Zoladek et al., 2007), while other receptors and other mediators of inflammation, may be induced after appropriate activation signals from other brain cells (Meeuwsen et al., 2003). Studies conducted by van Kralingen, found that a number of inflammatory cytokines were elevated in the CNS following injury. In turn, in various neurological conditions there are elevated levels of specific cytokines (in serum or CSF), correlating with poor results in neurological evaluations. These include TNFα and IL-1β, which have proven to affect the function of the blood-brain barrier. A secondary inflammatory response to IL-1β and TNFα, leads to astrocyte activation, being the long-term effect of these cytokines detrimental to the survival of astrocytes. This reveals a potential new target cell, which may help explain some of the negative effects of these cytokines on brain tissue during neuroinflammation (van Kralingen et al., 2013).

## Microglial cell

Microglia are widely distributed throughout the brain and spinal cord (Lawson et al., 1990). These cells can be found in brain, spinal cord, retina and optic nerve, but mainly in the hippocampus and substantia nigra (Venneti et al., 2009), and correspond to approximately 5–20% of the total population of glial cells in the CNS (Perry, 1998). These cells are considered as a representative of the immune system in the CNS, since they possess the ability to perform phagocytosis, release cytotoxic factors and behave as antigen presenting cells (Hanisch and Kettenmann, 2007).

Microglia plays a key role in embryonic development as they can secrete growth factors important for the formation of the CNS, and also contribute to the maturation, regeneration and neuronal plasticity. Furthermore, in their resting form they also are involved in other functions such as neurogenesis, neuroprotection and synaptic pruning, which has been found to be complement dependent (Sierra et al., 2010; Vinet et al., 2012). Moreover, these cells are also involved in a number of key processes for the maintenance of homeostasis of brain microenvironment, showing various functions. For example, microglia act as activated macrophages and they respond to any type of tissue injury (Nimmerjahn et al., 2005). Thus, the suitable and appropriate function of microglial cells is essential for the homeostasis of the CNS in both diseased and in normal health frame (Perry et al., 2010).

Microglia under physiological conditions are usually found in an inactivated state (or resting state) which is characterized by a ramified morphology, small and low expression of macrophage related molecules. When activated, drastic changes in morphology of microglia occurs. Activated microglia are not defined by a particular morphology, but are characterized by having few branchings, and a larger cell body with ameboidal form (Hanisch and Kettenmann, 2007).

Numerous signals represent a threat to the homeostasis of the CNS, including structures and/or residues from bacteria, viruses and fungi. Abnormal endogenous proteins, complement factors, antibodies, cytokines and chemokines, among others, are also sensed by the microglia elements and subsequently induce activation (Venneti et al., 2009). Thus, there are two main functional aspects of microglial cells: immune defense and maintenance of CNS homeostasis.

Activation of microglia by TLRs and NOD-like receptors (NLRs) is considered to be “classical” form of microglial activation where innate immune responses include production of proinflammatory cytokines like TNF-α, IL-1 and IL-6, and chemokines. Classical activation also leads to adaptive immune response by expressing major histocompatibility class II (MHCII) molecules and interaction with T cells (Olson and Miller, 2004). Under inflammatory conditions, there is an increase in active immune response and microglia should moderate the potential damage to the supporting tissues, repair and remodeling of the CNS (Ginhoux et al., 2013). In this state the cells regulate the expression of different surface markers, such as MHCII, growth factors (Harms et al., 2013), PPRs, produce more pro-inflammatory cytokines, such as IL–1β, IL-6, IL-12, interferon gamma (INF-γ) and TNF-α (Xiao et al., 1996). Moreover, activated microglia increase their proliferation (Venneti et al., 2009), synthesize and release cytotoxic factors such as superoxide radicals $\left(O_{2}^{-}\right)$, nitric oxide (NO) and reactive oxygen species (ROS) (Colton and Wilcock, 2010; Ha et al., 2012). Therefore, it becomes clear that microglials cell have an important role in innate immunity and are the main source of pro-inflammatory factors in brain.

Microglial activation is a phenotypically and functionally different process, since depending on the type, intensity and context of the stimulus; microglial response has a potential neuroprotective or pro-inflammatory effect (Hanisch and Kettenmann, 2007). It is precisely this delicate balance between the neurotoxic and neuroprotective and between pro-inflammatory and anti-inflammatory which determines the role of microglia in a disease or condition. So based on the current research, microglial activation should not be considered as an all or nothing event or single process, and we must realize that the answers to the pathological events depends on context and adapt as changes in the microenvironment occurs.

Studies by Nimmerjahn revealed that microglial processes and arborizations are highly mobile (Nimmerjahn et al., 2005). These are continuously being reconstructed by *de novo* formation and removal of processes, similar to the movements of the filopodia. Such a dynamic and thorough reorganization may allow microglia to fully explore their environment in any situation without disturbing the structures of near neurons. Thus, it is estimated that the entire brain parenchyma could be monitored in a few hours. This is possible because neighboring microglial cells take turns scanning shared regions, ensuring comprehensive detection, avoiding their own contact. This exploration generated by random processes that change quickly, can lead to the translation of microglial cells into a particular site induced for microdamage. Moreover, microglia cells also have many receptors for a large number of molecules, which can immediately detect signs of disruption in the structural and functional integrity of nervous tissue.

*In-vitro* studies have shown that microglial cells participate in the removal of Aβ peptide in culture (Hardy and Selkoe, 2002). But there is also possible that Aβ protofibrils activate microglia, triggering an inflammatory response and the subsequent release of neurotoxic cytokines. On the other hand, studies in patients receiving NSAIDs in long term treatment revealed a decrease in the incidence of AD, suggesting that attenuation of the inflammatory response may help prevent or maintain a lower probability of developing AD (McGeer et al., 1996, 2006; Fernández et al., 2008; Town, 2010). Even in mice, NSAIDs directly affect the development of amyloid in the brain, reducing Aβ-42 peptide levels (Weggen et al., 2001). This highlights the important role of pro-inflammatory cytokines released as a result of microglial activation and the effect of the damage signs, as major players in the development of AD (Figure 1).

In this context, authors Fernandez and Maccioni, have hypothesized the central role of neuroimmunomodulation in the pathogenesis of AD. In this model a number of innate damage signals, which manifest persistently over time, are able to induce microglial activation, triggering an overactivation state in the cells (Fernández et al., 2008; Maccioni et al., 2009; Morales et al., 2010).

**Figure 2:**
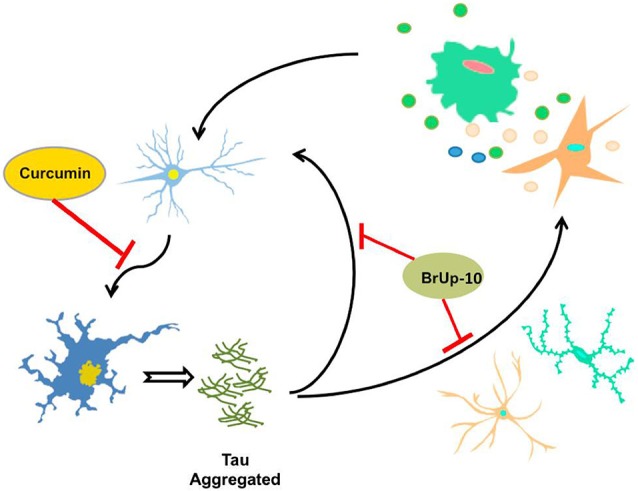
**τ aggregates potentiate the neuroinflammatory process**. Activated microglia and astrocytes, induce neuronal death through neuroinflammatory process, allowing the release of τ protein aggregates from the dead neuron. These aggregates of τ are capable of inducing activation of microglia and astrocytes, as well as neuronal death, thus producing a circuit of constant neuroinflammation. Considering the possible protective effects of curcumin, neuronal death could be delayed or inhibited by this compound, halting this circuit of neuroinflammation. Furthermore, the nutraceutical complex Brain Up-10 acts on τ aggregates, and may prevent neuronal death and activation of microglia and astrocytes, stopping the circuit and the development of neuroinflammation.

As prolonged exposure to damage signals generate overactivation of microglial cells, the steady release of cytotoxic factors and pro-inflammatory cytokines causes a neuroinflammatory phenomenon which is directly related to neuronal degeneration, mainly as a result of pro-inflammatory molecules (Li et al., 2007; Colton and Wilcock, 2010; Morales et al., 2010), positioning directly microglia and cytokines as key elements in the development of neurodegenerative disorders such as AD (Mrak, 2009; Figure 2).

Pathological τ aggregates are able to induce microglial activation with the subsequent events related to the neuroinflammatory cascade. After neuronal death, pathological τ protein aggregates are released into the extracellular medium causing activation of microglia and generating a cascade of constant feedback of damage signals (Morales et al., 2013).

## New research avenues

Today, as a result of the lack of effectiveness of current treatments for AD, a lot of effort has been invested to enhance the search for new therapeutic targets. Based on the results obtained in patients taking anti-inflammatory drugs, a new possibility has been opened studying the association of inflammatory processes and AD pathophysiology.

An important strategy to prevent brain impairment is based on dietary changes and nutritional supplements, functional foods and nutraceuticals. In this regard, there is interesting information coming from studies with the antioxidant and antiinflammatory *Andean Compound* (called initially as Andean Shilajit). *Andean Compound* is a very complex mixture of humic substances generated by natural millenary decomposition of vegetal material and is originated as an endemic natural product from the Andes Mountains. *Andean Compound* and its major active principle—fulvic acid—emerge as novel nutraceutical with potential uses against neurodegenerative brain disorders (Carrasco-Gallardo et al., 2012a). This natural compound is a potent anti-inflammatory substance, and a very safe dietary supplement (Carrasco-Gallardo et al., 2012b). In fact, according to studies by Cornejo et al. (2011), fulvic acid is able to block τ self-aggregation affecting the length and morphology of PHFs generated *in vitro*. Additionally, after exposure of preformed τ fibrils to fulvic acid, a decrease in length of PHFs can be detected (Cornejo et al., 2011).

Therapies based on τ protein, appear as an interesting target because tangle formation has been identified as a major event involved in the neurodegenerative process. Currently, our group is working in a compound that contains *Andean Compound* plus complex B vitamins (i.e., B6, B9 and B12 vitamins) named as Brain-Up 10®, underwent a pilot clinical trial and showed a tendency toward less cognitive deterioration, a reduction on neuropsychological symptoms and less distress for the caregiver of treated patients.

Another compound of natural origin, which is currently under study, is curcumin. Curcumin is a natural phenolic compound derived from the perennial herb *Curcuma longa* (turmeric), and is well known to exhibit anti-inflammatory and antioxidant activities (Aggarwal and Harikumar, 2009; Lu et al., 2014). In India, turmeric has traditionally been used for the treatment of diseases associated with injury and inflammation. The information about this compound reported that it may be capable of preventing the death of neurons in animal models of neurodegenerative disorders, but its possible effects on development and neuroplasticity are unknown (Kim et al., 2008). Studies led by the author Kim revealed that curcumin has a dual action in cell cultures of NPC (multi-potent neural progenitor cells): at low concentrations stimulates cell proliferation, whereas at high concentrations it becomes cytotoxic. On the other hand, the administration of curcumin to adult mice resulted in a significant increase in the number of newly generated cells in the dentate gyrus of hippocampus, indicating that this compound enhances adult hippocampal neurogenesis (Kim et al., 2008). Then, curcumin would stimulate developmental and adult hippocampal neurogenesis, with a biological activity that may improve neural plasticity and repair. Recent studies have shown that curcumin was able to prevent damage from Aβ, because it induced decrease in CaMKII function in organotypic hippocampal slices, attenuating synaptic dysfunction, inducing the development of more robust and synaptically efficient neurons, and this is reflected in the inhibition of synaptic dysfunction and neuronal death (Hoppe et al., 2013). These results expand the neuroprotective role of curcumin to a synaptic level, enhancing this compound as an alternative or add-on treatment for AD (Figure 2). In both examples—i.e., Brain-Up 10® and curcumin—we show that by looking at natural compounds it is possible to find new alternatives in the search of treatments for AD, so it is important not only to continue generating new synthetic compounds, but also to revisit old traditional medicine as well.

Other compounds have been used for supplementary treatment of AD (Table 2). *Ginkgo biloba* has been highly investigated but the data are confusing. Thus, Canevelli proposes that *Ginkgo biloba* may provide some cognitive benefits in AD patients, but only in cases under cholinesterase inhibitors treatment, but the clinical output of such effects remains to be clarified (Canevelli et al., 2014). On the other hand, in 2012 Vellas and coworkers conducted several clinical trials to study the effect of *Ginkgo biloba* extract in AD patients and healthy elderly subject that used this compound for longer periods of time. They aimed to assess the efficacy of 5 years’ administration of a standardized *Ginkgo biloba* extract, widely used as coadjuvant in the treatment of patients with cognitive disorders. However, the results failed to show a protective effect in this type of cognitive disorders (Vellas et al., 2012).

**Table 2:** **Natural Compounds recommended in the treatment AD**.

**Compound**	**Active Principle**	**References**
Ginkgo biloba	Ginkgo extract EGb761	Canevelli et al. (2014)
		Vellas et al. (2012)
Resveratrol	3,4,5-trihydroxystilbene	Lu et al. (2012)
		Kang et al. (2009)
Cerebrolysin	Porcine brain-derived peptide	Wei et al. (2007)
		Rockenstein et al. (2006)
		Álvarez and Fuentes (2011)

Resveratrol is a powerful antioxidant that is present in many plants, including grapes, peanuts and plums, that protects against environmental stress. This compound has been extensively investigated for their potential properties in cardioprotection, anti-inflammatory effects, anticancer, and antiaging effects. It was also shown recently that it inhibits Aβ aggregation *in vitro* (Lu et al., 2012). The problem is that it exhibits a low bioavailability in the organism (Kang et al., 2009). On the basis of knowledge that resveratrol possesses a variety of bioactivities, a novel series of derivatives have been generated and tested as multi-target agents for AD treatment (Lu et al., 2012).

Another compound used in AD treatment is Cerebrolysin. This compound is a neuropeptide preparation consisting of low-molecular-weight peptides and free amino acids. This compound mimics the action of endogenous neurotrophic factors, and it is postulated that a mixture of this peptide with neurotrophic effects may reduce neurodegenerative pathologies (Rockenstein et al., 2006). Wei et al. proposed that the main effects of Cerebrolysin include neurotrophic stimulation, neuroimmunological regulation and the improvement of glucose transportation across blood-brain barrier (Wei et al., 2007). After conducting a meta-analysis of six clinical trials, these authors, have postulated that Cerebrolysin could improve the clinical condition of AD patients, but large-scale trials are needed to provide convincing evidences of the efficacy of this compound on cognitive function and activities of daily living in AD (Wei et al., 2007). Further experimental research is needed to elucidate the molecular mechanism involved in some of the pleiotropic activities of Cerebrolysin, and particularly its influence on neuroinflammation, as well as to identify their possible interactions with neurotrophic factors and brain receptors (Álvarez and Fuentes, 2011). Main features of these agents are summarized in Table 2.

## Conclusion

The development of AD involves a series of perturbations and imbalances that systemically affect the normal functioning of the CNS, triggering a condition of dementia. Despite scientific advances and knowledge that exists regarding the AD, still it has not been possible to develop effective treatment options. As it is now, most available treatments are designed against AD symptoms, and serve only as palliative treatments. In this context, Neuroimmunomodulation hypothesis appears as a guide in the search for new targets that have not been considered before, for developing effective treatments. This is the case of τ protein, which under pathogenic conditions self-aggregates and becomes one of the most important actors in the neurodegenerative cascade.

Based on the results of studies on long-term exposure to anti-inflammatory drugs, that show that these drugs are associated with a decreased risk of developing AD, a new interesting therapy may be available. NSAIDs may protect people from Alzheimer through several potential mechanisms that are associated with the disease based on the neuroinflammatory theory (Akiyama et al., 2000; Szekely et al., 2004). For example, they can reduce the inflammatory processes in the brain, because these drugs can inhibit the inflammatory response of microglial and/or astrocytes, reducing cell death due to excitotoxicity mediated by glutamate (Casper et al., 2000).

But the use of these type of drugs requires definitely more exploration and analysis, especially of the mechanism of action leading to an improvement in the patient after prolonged use. The same applies to any neuroprotective effect, since this is a very sensitive issue that should be considered to estimate the effect of these drugs in the body.

The appearance of new compounds that can open the way to new treatments becomes a necessity. In this search we can mention compounds such as curcumin and Andean Compound, which, because of their natural origin and the lack of adverse effects, appears as promising preparation for AD prevention. Studies that have been made on these compounds are very recent, but give strong evidence that its effects are mediated by disruption of the inflammatory response and self-aggregation of the τ protein, positioning them as a future therapeutic option for neuronal injury.

## Conflict of interest statement

The authors declare that the research was conducted in the absence of any commercial or financial relationships that could be construed as a potential conflict of interest.
